# Linguistic complexity of EFL writing with different levels of English proficiency: A stratified study of an application-oriented university

**DOI:** 10.1371/journal.pone.0349399

**Published:** 2026-05-20

**Authors:** Jinhua Zhang

**Affiliations:** School of General Education, Xihang University, Xi’an‌‌, China; Universiti Sains Malaysia, MALAYSIA

## Abstract

English writing competence is a significant manifestation of students’ second language proficiency. However, comprehensive synchronic empirical research remains scarce regarding how students at application-oriented universities perform in terms of linguistic complexity in their compositions. To bridge this gap, this study analyzes 66 students’ compositions hierarchically at the level of lexis, syntax, and text. Results demonstrate that: (1) Lexical and syntactic complexity indicators exhibit different sensitivities to writing proficiency. (2) Both lexical and syntactic competence display non-linear developmental features. (3) Students’ text coherence is consistently correlated with the writing quality. Future research should prioritize students’ metalinguistic cognition towards different linguistic dimensions. EFL instruction should be tailored to students’ varying language proficiency levels. This study highlights the need for curricula that evolve alongside students’ English linguistic complexity development, offering new insights into English writing education at Chinese application-oriented universities.

## 1 Introduction

Among all language skills, writing is widely recognized as one of the most challenging tasks for foreign language learners [1,2]. As a crucial productive language skill, writing involves language proficiency, the learner’s comprehension of language, and critical thinking abilities throughout the writing process [3]. It is still a problem concerning the extent to which language proficiency interacts with and influences writing skill [4]. There are widespread concerns about EFL writing assessment and instruction [5–7], which encompasses the assessment and instruction in language, content, and organization [7,8]. Many standardized writing assessment rubrics contain measures in language, content, and organization [8,9], among which language proficiency is given priority by many standards [10,11]. Within this context, linguistic complexity has been widely considered as an objective, quantitative measure of learners’ language proficiency in L2 writing research [12]. Compared to human ratings, linguistic complexity could be measured objectively and efficiently. The assessment of linguistic complexity in student writing is of great importance for standardized testing [13].

Such objective assessment is especially needed in specific institutional settings, such as China’s application-oriented universities, which constitute a vital sector of the country’s higher education system. These institutions aim to cultivate application-oriented talents who serve as the intermediate force for the transformation of scientific research achievements [14]. Application-oriented talents are individuals who are engaged in serving economic and social development at the undergraduate level or above [14]. English writing competence, as a fundamental skill, plays a crucial role in the cultivation of application-oriented talents. However, many universities do not prioritize writing instruction adequately nor tailor it to their institutional type, educational objectives, or students’ actual learning conditions.

Process-based writing theory emphasizes writers, writing subjects, the process of text production, and assessment of writing text [15]. Focusing on the assessment of the writing text, the present study aims to reveal the writing language proficiency of students at application-oriented universities. A clear position is the prerequisite for the development of application-oriented universities [14]. In order to provide the targeted instruction, the priority is to understand the current situation of students’ English writing competence in such institutions, identify the main problems in writing competence among students at different levels of English proficiency, understand their reasons, and adopt corresponding teaching strategies. By addressing these questions, this study offers three distinctive contributions. First, this study extends prior work from research-oriented universities to application-oriented universities. Second, this study employs a synchronic stratified design to capture the current state of linguistic complexity across three proficiency levels simultaneously. Third, moving beyond the isolated examination of single linguistic dimensions, this study adopts a comprehensive evaluation framework encompassing lexical, syntactic, and textual levels, thereby providing a more systematic understanding of EFL writing competence. These contributions collectively inform proficiency-differentiated instructional strategies to the specific needs of application-oriented university students.

## 2 Literature review

### 2.1 Writing competence

Writing competence refers to learners’ ability to produce written texts in a foreign language using their language knowledge and writing skills [16]. “The ability to compose text from words is based on writing skills, and according to the fact that the product of written speech is written text” [16]. This competence requires support from various types of abilities [16]. English writing competence is a comprehensive reflection of language proficiency and has attracted global attention [17].

“Writing seems the most problematic skill as opposed to other ones since it requires creativity, language awareness, and critical thinking” [16]. However, in China, both from a pedagogical perspective and from a students’ perspective, English writing skill has not received special attention compared to other English language skills. In 2010, a survey conducted by the Ministry of Education on the current status of College English instruction in 530 universities nationwide found that participating institutions generally ranked the importance of English language skills as reading, listening, speaking, writing, and translation [18], indicating English writing skill is not valued by universities as much as listening and speaking in English instruction in China [19]. Years later, existing studies found that most students attribute their insufficient English writing competence simply to inadequate language proficiency, such as vocabulary deficiency, without comprehensively understanding deeper reasons for their English writing deficiencies [19,20]. Despite nearly a decade of teaching exploration [21–24], English writing instruction in Chinese universities is still under exploration, while the prerequisite for writing instruction is to know students’ writing competence, highlighting the urgency for a comprehensive study on English writing competence.

Enhancing English writing competence primarily involves understanding its components and evaluation criteria, which has been a topic of ongoing scholarly interest. Existing research has primarily focused on aspects such as language proficiency [25–27], critical thinking [28–30], and other dimensions to assess English writing competence [7,16]. Some scholars have selected variables such as lexical complexity, syntactic complexity, accuracy, and fluency to assess students’ English writing competence [23,26,31]. Liang [32] and Wang & Xie [33] evaluated learners’ written language quality based on discourse coherence. Previous studies did not reach a consensus about assessments of English writing competence, which have primarily focused on the individual variable in isolation rather than integration, and have yet to establish a systematic evaluation rubric for English writing competence. Thus, the present study aims to reveal students’ English writing competence in terms of language proficiency from a systematic evaluation rubric.

### 2.2 Linguistic complexity in writing

Linguistic complexity could be defined from both a conceptual perspective and an operational perspective. From the conceptual aspect, “linguistic complexity is defined as the capacity to use more advanced linguistic forms and functions” [12], which is equipped with multidimensional features. Considering its wide coverage of linguistic features, it could be used to measure learners’ language proficiency quantitatively. A growing number of studies in linguistic complexity of EFL writing have been conducted [25,34].

Because of the multidimensional nature of linguistic complexity, its operational definition varies from one study to another. The linguistic features of ESL/EFL writing have been explored from the lexical level and syntactic level most often [13,25]. Linguistic complexity features, especially syntactic complexity and word frequency measures, were confirmed to be related to the human rating of the language aspect of writing quality [13]. Based on previous studies, many studies have explored the predictive power of linguistic features over writing quality, and they have revealed the effective linguistic features, including lexical complexity, syntactic complexity, cohesion, and so on [25], which have already been proposed as main problems in students’ English writing [35]. Among the measures mentioned above, the lexical sophistication is confirmed to be an extremely strong predictor [36]. Syntactic complexity features, including length-based, clausal subordination, and phrasal complexity measures, are often explored in relation to language proficiency [12,13]. Both the lexical level and syntactic level have been prioritized over the discourse level in the previous studies [12,13]. However, the significance of discourse features in the EFL writing instruction and assessment was raised repeatedly [33,35]. Previous studies have indicated that “higher-rated writing tends to contain more frequent and diverse use of discourse organizational markers” [37].

Above all, referring to the research [12], the lexical level, the syntactic level, and the discourse level would be studied in the present research as they have been verified as effective measures in relation to learners’ language proficiency [38–41]. A comprehensive measure of linguistic complexity about EFL writings from these three aspects will reveal the participants’ language proficiency in EFL writing competence.

### 2.3 Assessment of writing quality

“In the assessment of EFL writing, language, content, and organization generally contribute to the overall quality of an essay and are accepted as the three major criteria in raters’ scoring decision-making process in both classroom and large-scale standardized assessment contexts” [9]. As the manifestation of writing assessment, the scoring methods, namely, including holistic and analytic scoring, are employed to present students’ writing competence assessment holistically and analytically. Many studies have confirmed that human raters are often concerned about language in scoring rather than organization [42,43]. Essay organization often refers to “transitions between paragraphs” and “deeper textual aspects such as the coherent flow of logical ideas” [44], which were indicated as related to writing quality [8].

The human raters’ holistic scoring behavior itself is a complex process, and it depends a lot on the rater’s personal reflection [45]. Thus, to improve the reliability of human raters, an authoritative analytic writing rubric would be provided to human raters. Thus, the holistic scoring could be turned into a combination of holistic scoring and analytic scoring, which could be seen as an effective proof of the writing quality of an essay. In the written language production, the linguistic features of a text are affected by various factors, including individual differences in language proficiency [46,47]. Different features in the written language production could be observed, which thus differentiate between proficiency levels [13]. The values of complexity measures highly depend on context [13]. By measuring the linguistic complexity of learners’ language performance, research on language proficiency has been conducted [13]. Thus, it is necessary to explore the individual differences of students’ language proficiency in the context of an application-oriented university.

Many scholars have criticized the phenomenon that most linguistic complexity-related studies have focused on the domains of syntax and lexis rather than implementing a more comprehensive approach [48]. In research on writing quality assessment, which studies how linguistic text characteristics relate to text quality, measures of text cohesion are added to the domains of syntax and lexis [49,50]. A relatively consistent assessment of writing quality has been reached, containing lexis, syntax, and discourse.

### 2.4 Instruction of EFL writing

It is necessary for writing teachers to ensure that their pedagogical choices are well matched with their students’ needs [51]. Many studies have advocated the importance of the context in designing the instruction [52,53]. Lack of consideration of the instructional context would result in an ineffective class, which fails to motivate the students and encourage their active participation in the class activities. In the writing instruction, teachers need to consider individual differences in the students’ language proficiency, which calls for stratified instruction for all the students with different levels of English proficiency. Some studies have explored the differences in students’ language proficiency in writing towards students in different language contexts [54–56]. The existing studies rarely cover students in an application-oriented university in China.

As indicated in many studies [57,58], there has been a lack of professional development in the teaching of writing, and teachers are largely unprepared to support the development of their students’ writing proficiency. This study was motivated by the lack of information about English writing proficiency of students in application-oriented universities. With the lack of research in the area, there has been little information about the linguistic features among high-, middle-, and low-proficiency students. Given the serious individual differences existing in the English classes [59], understanding the writing behaviors of the students at different English proficiency levels will be helpful for a more efficient development of the students’ English writing ability.

### 2.5 Research gap and the present study

The existing research on English writing competence has predominantly focused on various writing subjects, aiming to reveal their writing competence in lexis, syntax, discourse, and other factors. However, there are the following deficiencies: (1) From the research context: a limited range of research context were concentrated. Research on the writing subjects has primarily centered on different age groups and different majors, with a few studies specifically examining the writing competence of graduate students [21], students in secondary school [13], students in the English major [60,61], and students in the engineering major [23]. The exploration of students from an application-oriented university is sparse. (2) From the research perspectives: In recent years, a lack of synchronic research regarding writing competence exists. Studies on writing competence have primarily explored its diachronic dynamic development [23,26], with limited empirical research on current synchronous states. (3) From the research content: Existing studies have not yet developed a systematic evaluation of students’ writing competence, and evaluation criteria are often one-sided and inconsistent. Assessments of English writing competence have primarily focused on the individual variable in isolation rather than integration, which needs to reach a consensus about systematic assessments of writing competence. (4) From the practical significance, there has been a lack of professional norms in the writing instruction, and teachers are largely unprepared to support the development of students’ writing proficiency. Furthermore, there is a lack of information about linguistic features among students with different language proficiency. With regard to the big individual differences in the English classes, it is necessary to understand students’ writing performances and differences, which will be beneficial to students’ efficient development of English writing competence and to English writing instruction.

To sum up, these limitations call for the present study by focusing on students from application-oriented universities and conducting a synchronic study on their English writing competence. To fill this gap, the present study will systematically evaluate students’ writing competence, with a particular emphasis on language complexity at the lexical level, syntactic level, and discourse level. The present study aims to address the following two research questions:

(1) How do students at different levels of English proficiency perform in terms of lexis, syntax, and text in their compositions?
(2) What teaching strategies are necessary to improve the writing ability of students in an application-oriented university?

## 3 Methods

### 3.1 Research method and data collection

This study employs a corpus analysis method. Research is designed upon existing literature and is not related to human participants’ privacy. A formal waiver was granted by the academic committee. In preparing the data for answering the research questions, all the essays were processed with corpus analysis tools. The essay data are sourced from 66 compositions written by college students who participated in an English writing competition at an application-oriented university in China. These data do not contain any information that can identify an individual. Therefore, ethical approval is exempted. The requirement for informed consent was waived because this study was based on pre-existing writings collected from an English writing contest. All the participants in this research are Chinese undergraduates with Chinese as their native language and English as their foreign language. They have learned English for more than 10 years. All of them have participated in the English test in the college entrance examination in China. Compositions were written within 60 minutes with the same given topic: cloud tourism. The writing topic was selected because it aligns with the contemporary sociocultural context and is familiar to all participants, thereby minimizing topic-related variability. This topic is also representative of the types of argumentative writing tasks commonly encountered in College English instruction and assessment in application-oriented universities, enhancing the ecological validity of the findings. A corpus was constructed with a total of 22,256 tokens.

After the competition, all the essays were rated by two experienced experts with the writing rubric of College English Test Band 4, with a maximum score of 15 points. The inter-rater reliability, as indicated by a high intraclass correlation coefficient (r > 0.8), demonstrated strong consistency and reliability. Consequently, the mean scores assigned by the two raters were adopted for subsequent analysis. To facilitate a comparative analysis of the language complexity indices across groups of a comparable size, the essays were divided into three proficiency groups with an equal number--high-, middle-, and low-score groups based on the score with 22 essays each. The equal size stratification follows established practice in L2 writing research: it maximizes statistical power for group comparisons and is a recognized sampling strategy in corpus design [62]. The pairwise comparison among three groups indicated the significant difference between each pair of groups. The score distribution across these groups aligned substantially with their respective performance levels. The constructed corpus is presented in Table 1.

**Table 1:** Information of self-built writing corpus.

Groups	Topic	Texts number	Tokens	Types	Average texts length	Max/Min text length	SD
High-score	Cloud Tourism	22	10617	1565	480	546/385	46.91
Middle-score	Cloud Tourism	22	7983	1155	358	517/252	95
Low-score	Cloud Tourism	22	3656	685	164	324/31	66.92
Total		66	22,256	2157			

After confirming the normality of mean scores among three groups, a One-way ANOVA was performed. The homogeneity of variances in mean score was equal (*p* = 0.603), indicating that the group variances were equal in mean score. Post hoc multiple comparisons revealed statistically significant differences in mean scores between the high-score and middle-score groups (*p* = 0.000, *η*^*2*^ = 0.885), as well as between the middle-score and low-score groups (*p* = 0.000， *η2* = 0.885). The effect size was very large (*η2* = 0.885). A text cleaning tool, “Text Organizer,” was used to clean and manually segment the compositions. This procedure involved removing extra spaces, handling mixed use of Chinese and English punctuation, eliminating garbled text, removing redundant blank lines, and manually segmenting cases where multiple words were connected without spaces. The cleaned text was then put into analysis.

### 3.2 Data analysis

In order to answer the research questions, this study will analyze the compositions in high-score, middle-score, and low-score groups from lexis, syntax, and texts. In terms of vocabulary, lexical richness is used to measure the effective use of vocabulary. A composition can be assessed with the following dimensions: lexical variation or type-token ratio, lexical sophistication, lexical density, and the number of errors [63]. It is hard to define word errors precisely, and the severity of different types of errors needs to be considered [63]. Therefore, this study will not consider word errors, and it will explore the linguistic performance of students’ compositions in different groups by examining word length, lexical variation, lexical sophistication, word frequency distribution, and lexical density, which is aligned with the indices used by Hao et al. [64]. In preparing the data for measuring lexical variation (also labeled as lexical diversity), all the essays were processed with Wordsmith 8.0, Lexical complexity analyzer and Coh-Metrix to yield basic quantitative data about standard type-token ratio (STTR) (i.e., lexical variation), word length and LDMTLD, with Range 32 and lexical complexity analyzer to measure lexical sophistication and word frequency distribution, and with lexical complexity analyzer to measure lexical density. The data analysis will be conducted with Wordsmith 8.0, Range 32, Lexical complexity analyzer, and SPSS 26.0 will be used for comparative tests between groups. Wordsmith 8.0 is a powerful corpus analysis tool; Range 32 is a powerful text analysis tool to process the word frequency distribution; Lexical complexity analyzer is a comprehensive lexical analyzing tool; and Coh-Metrix is an automated analysis tool of the language and discourse characteristics of texts.

From the perspective of the syntactic dimension, syntactic complexity, aligning with other authoritative studies [65,66], is categorized into unit length and clausal density. The unit length is measured by T-unit length and clause length, and clause density is measured by T-unit complexity ratio and dependent clause ratio [67]. T-unit length is average word count per T-unit (W/T); clause length is the average word count per clause (W/C). T-unit complexity ratio measures clause count per T-unit (C/T); Dependent clause ratio measures dependent clause count per clause (DC/C). These two categories have different focuses: length emphasizes the breadth or range of syntactic complexity, while clausal density emphasizes the depth or subordination of syntactic complexity. Besides this, average sentence length and sentence syntactic similarity will also be considered. The unit length and clause density are calculated with the L2 Syntactical Complexity Analyzer, and the sentence syntactic similarity values are calculated with Coh-Metrix 3.0. L2 Syntactic Complexity Analyzer (L2SCA) was designed to automate syntactic complexity analysis of L2 English texts using 14 measures [68]. Coh-Metrix 3.0 is a computational linguistic tool, which was adopted in the research to measure multiple language features, including lexis, syntax, text, and so on [69]. It could be used to measure syntax, such as syntactic similarity [70]. L2 Syntactical Complexity Analyzer and Coh-Metrix have been verified effectively in many empirical studies [68,71,72].

In terms of texts, Coh-Metrix 3.0 is used for text analysis to obtain local cohesion variables and overall cohesion variables of the composition, which is aligned with the other study [32]. The analysis is conducted from two aspects: surface-level text feature variables and deep-level text feature variables, exploring the use of surface-level cohesion devices and the performance of deep-level coherence in each group of compositions.

In summary, the selection of linguistic complexity indicators was guided by both theoretical considerations and empirical precedents. At the lexical level, lexical variation (STTR, LDMTLD), lexical sophistication (LS1, LS2), and lexical density were chosen because they have been consistently validated as reliable predictors of writing quality in L2 contexts [39,63,73]. At the syntactic level, unit length (T-unit length, clause length) and clausal density (T-unit complexity ratio, dependent clause ratio) were adopted following Ortega’s [67] foundational framework, which distinguishes between the breadth and depth of syntactic complexity. At the textual level, local and overall coherence variables from Coh-Metrix were employed, as they capture both surface-level cohesive devices and deep-level semantic coherence, addressing a frequently overlooked dimension in L2 writing research [32,74].

## 4 Research results

In order to answer the first research question, the research results of the language complexity are demonstrated below.

### 4.1 Lexis

According to the book *Assessing Vocabulary* [63], this study is conducted from four dimensions in the lexis: lexical variation, word frequency distribution, lexical sophistication, and lexical density.

#### 4.1.1 Lexical variation.

The analysis results of 66 students’ compositions show that the standard type-token ratios for students’ compositions in high-score, middle-score, and low-score groups are 35.73%, 34.34%, and 31.77% (as shown in Table 2). In order to ensure accuracy, the lexical variation measure LDMTLD from Coh-Metrix 3.0 is employed in the pairwise comparison among groups (Table 3).

**Table 2:** Standard type/token ratio.

	High-score	middle-score	Low-score	Total
Token	10,617	7,983	3,656	22,256
Type	1,565	1,155	685	2,157
STTR	35.73%	34.34%	31.77%	34.42%
Average word length	4.63	4.36	4.49	4.51

**Table 3:** Lexical diversity: Measure of textual lexical diversity(LDMTLD).

	mean	max	min	SD
High-score group	77.07	118.284	48.337	19.34
middle-score group	78.71	125.399	53.678	17.48
Low-score group	79.67	269.080	38.180	47.31

Since the assumption of normality was violated for the lexical variation measure (LDMTLD) in the low-score group, a Kruskal-Wallis test was employed. The result did not reveal a statistically significant difference in the lexical variation, as measured by LDMTLD, among three groups (*H*(2)=1.614, *p* = 0.446).

#### 4.1.2 Words distribution.

By analyzing the writing texts from high-score, middle-score, and low-score groups using Range 32, their vocabulary distribution was retrieved. The specific vocabulary frequency distribution is presented in  Tables 4–6. Analysis reveals that students across three groups relied predominantly on the most commonly used 1000 words in their writing texts. The usage rates for words in Word List 1 are 58.16% in the high-score group, 66.72% in the middle-score group, and 70.16% in the low-score group. Notably, lower-proficiency writers exhibited heavier dependence on the most commonly used 1000 words (70.16%) alongside reduced employment of second high-frequency words in Word List 2 and off-list words.

**Table 4:** Words frequency distribution in high-score group.

WORD LIST	TOKENS/%	TYPES/%	FAMILIES
one	8666/81.63	910/58.16	567
two	1239/11.68	321/20.54	249
three	177/1.67	91/5.80	82
not in the lists	535/5.04	243/15.50	?????
Total	10617	1565	898

**Table 5:** Words frequency distribution in middle-score group.

WORD LIST	TOKENS/%	TYPES/%	FAMILIES
one	6849/85.80	771/66.72	506
two	705/8.83	195/16.94	164
three	101/1.26	68/5.85	63
not in the lists	328/4.11	121/10.49	?????
Total	7983	1155	733

**Table 6:** Words frequency distribution in low-score group.

WORD LIST	TOKENS/%	TYPES/%	FAMILIES
one	3012/82.37	481/70.16	360
two	405/11.08	104/15.28	90
three	63/1.73	37/5.39	34
not in the lists	176/4.82	63/9.17	?????
Total	3656	685	484

As evidenced by the lexical analysis (shown in the tables above), it can be seen that students use more high-frequency words from Word List 1 and fewer words from Word List 2 and Word List 3. The words that are absent from the word list account for a higher proportion than those in Word List 3. Examining those off-list words, it is found that they have a strong association with contemporary sociocultural issues. For instance, a large number of words such as COVID-19 and other relevant terms, as well as emerging social phenomena such as “live broadcasting” and “online celebrity”, digital media such as “APP” and “TikTok”, as well as personal names and geographical references such as “Zhong Nanshan” and “Mount Taishan”. Due to limited lexical proficiency, students frequently resort to pinyin transliterations for conceptually complex referents, contributing to the higher proportion of off-list words.

#### 4.1.3 Lexical sophistication.

Lexical sophistication is operationally defined as the ratio of low-frequency lexical items to total word count in a text [73]. Previous studies across linguistic contexts, including Du and Cai’s study [74], have consistently demonstrated its predictive validity for writing proficiency assessment. In order to quantify this construct, the study employs Range 32, which divides text vocabulary into four word lists: the most commonly used 1000 words, the second most commonly used 1001–2000 words, academic vocabulary, and vocabulary out of the three word lists. Following established methodology [39], lexical sophistication in this study is operationally defined as the proportion of academic vocabulary and off-list vocabulary to the total vocabulary.

As demonstrated in Table 7, it can be seen that low-frequency word types constituted 21.30% in the high-score group, 16.34% in the middle-score group, and 14.56% in the low-score group. This declining trend suggests a positive association between writing quality and lexical sophistication.

**Table 7:** Types and proportion of low-frequency words in three groups.

WORD LIST	High-score group type/proportion%	middle-score group type/proportion%	low-score group type/proportion%
three	91/5.80	68/5.85	37/5.39
not in the lists	243/15.50	121/10.49	63/9.17
Total	334/21.30	189/16.34	100/14.56

To compare lexical sophistication among the three groups, two indices, LS1 (lexical sophistication 1) and LS2 (lexical sophistication 2), from the lexical complexity analyzer were employed. Since the assumption of normality was violated for LS1 in the middle-score group, a Kruskal-Wallis test was conducted. The result revealed a statistically significant difference in LS1, among three groups (*H*(2)=6.155, *p* = 0.046). Post-hoc pairwise comparisons were performed using Mann-Whitney *U* tests with Bonferroni correction for multiple comparisons. The results showed the significant difference between high-score and middle-score groups (*U* = 132, *p* = 0.01, Bonferroni-adjusted *p* = 0.03, *r* = 0.39), indicating a medium effect size. No significant differences were observed between the middle-score and low-score groups (*U* = 310, *p* = 0.110, Bonferroni-adjusted *p* = 0.330, *r* = 0.24) and between high-score and low-score groups (*U* = 230.5, *p* = 0.787, Bonferroni-adjusted *p* = 1.000, *r* = 0.04).

For LS2, after confirming the normality of LS2 in three groups, a one-way ANOVA was performed. Levene’s test for homogeneity of variances in LS2 was significant (*p* = 0.049). Therefore, the more robust Welch’s ANOVA was reported. The Welch ANOVA revealed a statistically significant difference in LS2 among three groups, Welch’s *F* (2, 40.489)=4.37, *p* = 0.019, *ω*^*2*^ = 0.055, representing a medium effect size. Post hoc analysis using Games-Howell test indicated that LS2 in high-score group (M = 0.2164, SD = 0.03761) is significantly larger than that in middle-score group (M = 0.1850, SD = 0.03233), with a mean difference of 0.03136 (95% CI[0.0057, 0.0571], *p* = 0.014, Bonferroni-adjusted *p* = 0.042). The effect size, calculated as Hedges’ g using the standard deviation of the middle-score group, is 0.97, which is considered large. No statistically significant difference in LS2 was found between middle-score group and low-score group (M = 0.1945, SD = 0.05492) (Mean difference = −0.00955, 95% CI[−0.0428, 0.0238], *p* = 0.764, Bonferroni-adjusted *p* = 1). The effect size, calculated as Hedges’ g using the standard deviation of the middle-score group, is 0.30, which is considered a small-to-medium effect size. And no significant difference in LS2 was found between the high-score group and the low-score group (Mean difference = 0.02182, 95% CI[−0.0128, 0.0565], *p* = 0.285, Bonferroni-adjusted *p* = 0.855). The effect size, calculated as Hedges’ g using the standard deviation of the low-score group, is 0.40, which falls between small (*g* = 0.20) and medium (*g* = 0.50).

#### 4.1.4 Lexical density.

Lexical density, defined as the proportion of content words relative to total word count, serves as a key indicator of textual formality, and the higher the lexical density is, the higher the degree of the written form is [63]. Lexical density measures the ratio of content words to the overall words in the text and examines the information content of the text [75,76]. Ure [76] pioneered this operational standard and revealed that the ratio of content words in written texts typically exceeds 40%, while in spoken discourse, this ratio is below 40%. Empirically, lexical density correlates positively with formality of written English [63,77]. Formal registers exhibit higher density, whereas informal styles approximate spoken norms with reduced lexical density [77]. The lexical density values are demonstrated in Table 8.

**Table 8:** Lexical density.

Groups	Mean	Max	Min	SD
High-score group	0.4786	0.56	0.40	0.04212
middle-score group	0.4732	0.54	0.40	0.03859
Low-score group	0.5005	0.68	0.42	0.06176

The measurement results above show that the lexical density exceeded 40% in all three groups, confirming students’ capacity to differentiate written registers from spoken norms. To compare lexical density(LD) among the three groups, LD calculated by the lexical complexity analyzer is employed to conduct a pairwise comparison. The normality of LD was confirmed, and then a one-way ANOVA was performed. Levene’s test for homogeneity of variances in LD was not significant (*p* = 0.105). The ANOVA revealed no significant difference in lexical density among the three groups, *F*(2, 63)=1.942, *p* = 0.152, *η^2^* = 0.058, indicating a medium effect size. This indicates that the lexical density of compositions in this study does not present a significant difference, yet it still demonstrates the potential research significance.

#### 4.1.5 Summary.

In conclusion, the research results of lexis are summarized as follows (Table 9). Lexical sophistication emerged as a crucial indicator to distinguish the varying writing quality, exhibiting a non-linear developmental feature: the high-score group demonstrated markedly higher sophistication, while the middle-score and low-score groups showed comparable, lower levels. Students across three groups relied predominantly on the most commonly used 1000 words, indicating limited lexical repertoire. Overall, the results suggest a possible plateau in lexical complexity development among EFL learners at application-oriented universities. Although the middle-score group slightly outperformed the low-score group on several lexical indices, the difference did not reach statistical significance.

**Table 9:** Results of lexis among the three groups.

Indicators Pairwise comparison	Lexical variation	Lexical sophistication		Lexical density
		LS1	LS2	
High-score vs. Middle-score	**–**	**+**	**+**	**–**
Middle-score vs. Low-score	**–**	**–**	**–**	**–**
High-score vs. Low-score	**–**	**–**	**–**	**–**

+ represents a significant difference.

- represents no significant difference.

### 4.2 Syntax

Syntactic complexity had a significant predictive power on writing performance through the text analysis of native English speakers’ writings [71]. This section evaluates the syntactic complexity of the compositions through several metrics: mean sentence length, syntactic similarity, unit length, and clausal density.

#### 4.2.1 Sentence length and syntactic similarity.

The mean sentence length correlated positively with writing performance, while two syntactic similarity indices correlated negatively with writing performance [74]. The descriptive statistics on sentence length and syntactic similarity across the three groups are presented in Table 10.

**Table 10:** Sentence length and syntactic similarity value.

Groups	Tokens	The number of sentences	mean sentence length	mean syntactic similarity value
High-score group	10,616	545	19.48	0.0962
middle-score group	7981	446	17.89	0.1033
Low-score group	3657	255	14.34	0.1156
Total	22,254	1,246	17.86	

Regarding mean sentence length (MLS), a Kruskal-Wallis test revealed a statistically significant difference in MLS among three groups (*H*(2)=12.396, *p* = 0.002). Post-hoc pairwise comparisons were performed using Mann-Whitney *U* tests with Bonferroni correction for multiple comparisons. The results showed the significant difference between high-score and middle-score groups (*U* = 132, *p* = 0.01, Bonferroni-adjusted *p* = 0.03, *r* = 0.39), indicating a medium-to-large effect size. Significant differences were observed between the high-score and low-score groups (*U* = 91.5, *p* = 0.000, Bonferroni-adjusted *p* = 0.000, *r* = 0.53), indicating a large effect size. The difference between middle-score and low-score groups did not reach significant after correction (*U* = 145.5, *p* = 0.023, Bonferroni-adjusted *p* = 0.069, *r* = 0.34), indicating a medium effect size.

Since the assumptions of normality were violated in the low-score group, a Kruskal-Wallis test was conducted. The results revealed a statistically significant difference in SYNSTRUTt among three groups (*H*(2)=8.582, *p* = 0.014). Post-hoc pairwise comparisons were performed using Mann-Whitney *U* tests with Bonferroni correction for multiple comparisons. The results showed a significant difference between high-score and low-score groups (*U* = 370, *p* = 0.003, Bonferroni-adjusted *p* = 0.009, *r* = 0.453), indicating a medium-to-large effect size. No significant difference was observed between the high-score and the middle-score groups (*U* = 287, *p* = 0.291, Bonferroni-adjusted *p* = 0.873, *r* = 0.159), indicating a small-to-medium effect size. No significant difference between middle-score and low-score groups was observed between the middle-score and the low-score groups (*U* = 311.5, *p* = 0.103, Bonferroni-adjusted *p* = 0.309, *r* = 0.246), indicating a small-to-medium effect size.

#### 4.2.2 Unit length and clausal density.

Since the assumption of normality was violated in the T-unit length, a Kruskal-Wallis test was conducted. The results revealed statistically significant differences in T-unit length across three groups (*H*(2)=15.467, *p* = 0.000*). Post-hoc pairwise comparisons were performed using Mann-Whitney *U* tests with Bonferroni correction for multiple comparisons. The results showed no significant difference between high-score and middle-score groups after Bonferroni correction (*U* = 148, *p* = 0.027, Bonferroni-adjusted *p* = 0.081, *r* = 0.33), indicating a medium effect size. Significant differences were observed between the high-score and low-score groups (*U* = 78, *p* = 0.000*, Bonferroni-adjusted *p* = 0.000*, *r* = 0.58), indicating a large effect size. The comparison between the middle-score and low-score groups yielded a non-significant result after correction (*U* = 164, *p* = 0.067, Bonferroni-adjusted *p* = 1.000, *r* = 0.276), indicating a small-to-medium effect size.

Regarding clause length, since the assumption of normality was violated in clause length, a Kruskal-Wallis test was employed. The results revealed statistically significant differences in clause length (*H*(2)=16.967, *p* = 0.000*). Post-hoc pairwise comparisons were performed using Mann-Whitney *U* tests with Bonferroni correction for multiple comparisons. The results showed a significant difference between the high-score and middle-score groups after Bonferroni correction (*U* = 63, *p* = 0.000*, Bonferroni-adjusted *p* = 0.000*, *r* = 0.63), indicating a large effect size. Significant differences were observed between the high-score and low-score groups (*U* = 137, *p* = 0.014, Bonferroni-adjusted *p* = 0.042, *r* = 0.37), indicating a medium-to-large effect size. No significant difference was observed between the middle-score and low-score groups (*U* = 296, *p* = 0.205, Bonferroni-adjusted *p* = 0.615, *r* = 0.19), indicating a small effect size.

Clause density was examined using both the T-unit complexity ratio and the dependent clause ratio. Since the assumption of the T-unit complexity ratio was violated, a Kruskal-Wallis test was conducted. The results revealed statistically significant differences in T-unit complexity ratio(*H*(2)=8.228, *p* = 0.016). Post-hoc pairwise comparisons were performed using Mann-Whitney U tests with Bonferroni correction for multiple comparisons. No significant difference was observed between the high-score and middle-score groups(*U* = 272, *p* = 0.481, Bonferroni-adjusted *p* = 1, *r* = 0.11), indicating a small effect size. Significant differences were found between the high-score group and the low-score group (*U* = 127.5, *p* = 0.007, Bonferroni-adjusted *p* = 0.021, *r* = 0.405), indicating a medium-to-large effect size. No significant difference was found between the middle-score group and the low-score group after Bonferroni correction (U = 147, *p* = 0.026, Bonferroni-adjusted *p* = 0.078, *r* = 0.336), indicating a medium effect size.

Considering the dependent clause ratio, after confirming the normality of the dependent clause ratio among the three groups, a one-way ANOVA was performed to examine the differences across the three groups. Levene’s test for homogeneity of variances was not significant (*p* = 0.073), indicating that the group variances were equal. The result of a one-way ANOVA revealed no significant difference among the three groups (*F*(2,63)=1.367, *p* = 0.262, *η^2^* = 0.042), indicating a small-to-medium effect size.

#### 4.2.3 Summary.

In conclusion, the research results of syntax are summarized as follows (Table 11). Overall, the syntactic complexity effectively distinguished writing proficiency across the three groups. The high-score group outperformed the low-score group in most indicators, while no significant differences were observed between the middle-score and low-score groups on any indicator. The most distinguishing indicators were MSL and clause length, followed by syntactic similarity, T-unit length, and T-unit complexity ratio.

**Table 11:** Results of syntax among the three groups.

Indicators Pairwise comparison	MSL	Syntactic similarity value	Unit length		Clausal density	
			T-unit length	Clause length	T-unit complexity ratio	dependent clause ratio
High-score vs. Middle-score	**+**	**–**	**–**	**+**	**–**	**–**
Middle-score vs. Low-score	**–**	**–**	**–**	**–**	**–**	**–**
High-score vs. Low-score	**+**	**+**	**+**	**+**	**+**	**–**

+ represents a significant difference.

- represents no significant difference.

### 4.3 Text

The textual coherence is an important criterion for measuring the quality of a text. Textual coherence encompasses two aspects: form and meaning. The former refers to cohesive devices such as pronouns and conjunctions used on the surface of the text, while the latter refers to the continuity of ideas and the correlation of concepts [74]. Therefore, the present study employs the discourse coherence analysis system Coh-Metrix 3.0 to analyze the coherence of students’ writing texts. Coh-Metrix 3.0 extracts multiple textual indices, employing latent semantic analysis (LSA) -- a computational linguistics method to quantify textual coherence beyond superficial cohesive devices. This method can effectively analyze the coherence of a text without relying on surface features and explore the semantic correlation among various parts of the text [32].

Sixty-six students’ compositions were analyzed one by one with Coh-Metrix 3.0 to extract variables closely related to local coherence and overall coherence. They are local coherence variables, mainly involving various textual coherence features such as reference, substitution, and connection. There are a total of 14 variables, including adjacent argument overlap, adjacent stem overlap, LSA between adjacent sentences, personal pronouns, and various conjunctions. There are four overall coherence variables, which refer to argument overlap, stem overlap, LSA among all sentences, and LSA between paragraphs [32]. Among them, local coherence variables mainly refer to the characteristics of cohesive devices on the surface of the text. Liang [32] found that only 3 out of 14 variables (i.e., adjacent argument overlap, adjacent stem overlap, and LSA between adjacent sentences) have certain predictive power to writing grades, and 13 out of these 14 variables are different cohesive devices.

#### 4.3.1 Local coherence variables.

Local coherence variables of compositions in different groups are shown in the following tables.

**Fig 1:**
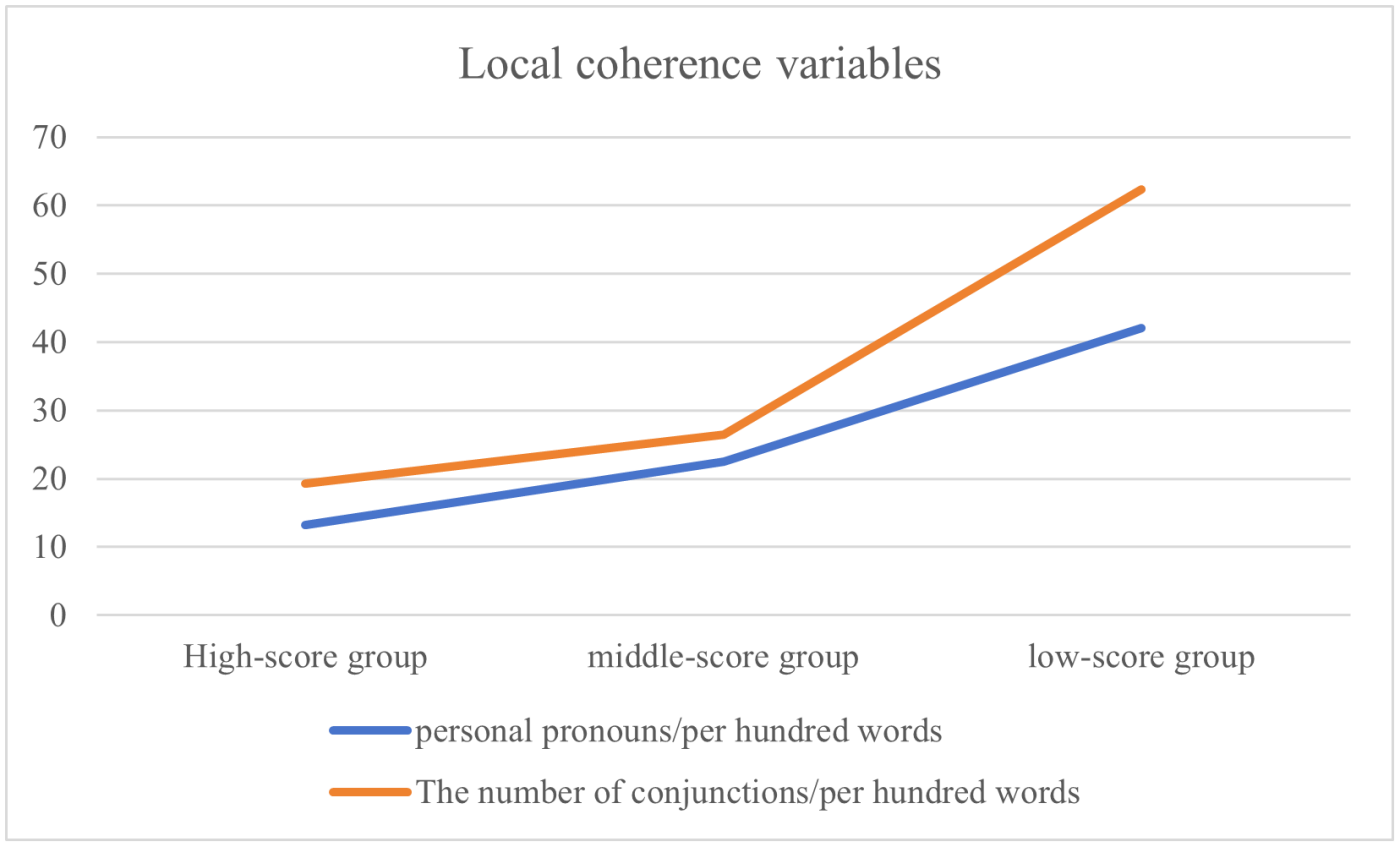
Local coherence variables.

Tables 12–15 show the performance of local coherence variables across proficiency groups, with personal pronouns and conjunctions representing the most prevalent surface cohesive devices in the text. To enable objective cross-group comparison of the frequency of cohesive devices, raw counts were normalized per hundred words due to significant differences in composition length. Analysis reveals an inverse relationship between writing scores and personal pronoun density: the high-score group used 13.20 pronouns per hundred words, compared to 22.42 in the middle-score group and 42.05 in the low-score group. This progression indicates that pronoun frequency increases as scores decrease (Fig 1). Given that personal pronouns exhibited a clear tendency of orality in written discourse [78], their frequent use may lead to the orality of the text [32]. Consequently, low-score compositions demonstrate increasingly pronounced oral characteristics. A similar tendency emerged for conjunction density: the high-score group used 19.27 conjunctions per hundred words, the middle-score group used 26.46 conjunctions per hundred words, and the low-score group used 62.35 conjunctions per hundred words, confirming that conjunction frequency likewise increases as scores decrease (Fig 1). In summary, these findings indicate that higher frequencies of surface cohesive devices correlate with poorer writing performance. The divergence between the high-score group and the middle-score group is smaller than that between the middle-score group and the low-score group.

**Table 12:** Local coherence variables in students’ compositions.

cohesive devices	High-score group	middle-score group	Low-score group
adjacent argument overlap	0.532455	0.550818	0.477636
adjacent stem overlap	0.438455	0.383409	0.334545
LSA between adjacent sentences	0.162682	0.142227	0.144136
personal pronouns	63.470318	80.251227	68.967318
personal pronouns/per hundred words	13.20	22.42	42.05
The number of conjunctions	92.6904	94.744227	102.261818
The number of conjunctions/per hundred words	19.27	26.46	62.35

**Table 13:** Local coherence variables in high-score group.

Cohesive devices	Maximum	Minimum	Mean	SD
adjacent argument overlap	0.866	0.293	0.532455	0.1583052
adjacent stem overlap	0.866	0.1750	0.438455	0.167177
LSA between adjacent sentences	0.3610	0.0790	0.162682	0.0612461
personal pronouns	122.6050	17.7160	63.470318	27.4845451
Total number of various conjunctions	117.6470	61.5070	92.6904	16.3976

**Table 14:** Local coherence variables in middle-score group.

Cohesive devices	Maximum	Minimum	Mean	SD
adjacent argument overlap	0.8750	0.2190	0.550818	0.1517252
adjacent stem overlap	0.6430	0.0450	0.383409	0.1695230
LSA between adjacent sentences	0.2610	0.0520	0.142227	0.0552164
personal pronouns	125.0000	35.2930	80.251227	24.1159832
Total number of various conjunctions	119.6910	59.3750	94.744227	18.1613391

**Table 15:** Local coherence variables in low-score group.

Cohesive devices			Maximum	Minimum	Mean	SD
adjacent argument overlap			0.8460	0	0.477636	0.2108266
adjacent stem overlap			0.8000	0	0.334545	0.1994862
LSA between adjacent sentences			0.3170	0	0.144136	0.0907300
personal pronouns			138.4620	0	68.967318	36.4913211
Total number of various conjunctions			133.3320	62.5000	102.261818	16.3075315

#### 4.3.2 Overall coherence variables.

Overall coherence variables of compositions in each group are shown in the  Tables 16–19.

**Table 16:** Overall coherence variables in students’ compositions.

Cohesive devices	High-score group	middle-score group	Low-score group
argument overlap	0.461682	0.4387273	0.4341364
stem overlap	0.408545	0.334	0.3234545
LSA among all sentences	0.15713636	0.1322273	0.1242727
LSA between paragraphs	0.44181818	0.3229091	0.3360455

**Table 17:** Overall coherence variables in the high-score group of compositions.

Cohesive devices	Maximum	Minimum	Mean	SD
argument overlap	0.732	0.280	0.461682	0.1323025
stem overlap	0.742	0.163	0.408545	0.1382343
LSA among all sentences	0.298	0.083	0.15713636	0.051742306
LSA between paragraphs	0.630	0.165	0.44181818	0.126409251

**Table 18:** Overall coherence variables in the middle-score group of compositions.

Cohesive devices	Maximum	Minimum	Mean	SD
argument overlap	0.732	0.118	0.4387273	0.13464740
stem overlap	0.609	0.034	0.334	0.15595634
LSA among all sentences	0.264	0.041	0.1322273	0.05889533
LSA between paragraphs	0.602	0.00	0.3229091	0.16208226

**Table 19:** Overall coherence variables in the low-score group of compositions.

Cohesive devices	Maximum	Minimum	Mean	SD
argument overlap	0.787	0.00	0.4341364	0.17349360
stem overlap	0.666	0.00	0.3234545	0.19056076
LSA among all sentences	0305	0.00	0.1242727	0.08171214
LSA between paragraphs	0.638	−0.008	0.3360455	0.22468888

**Fig 2:**
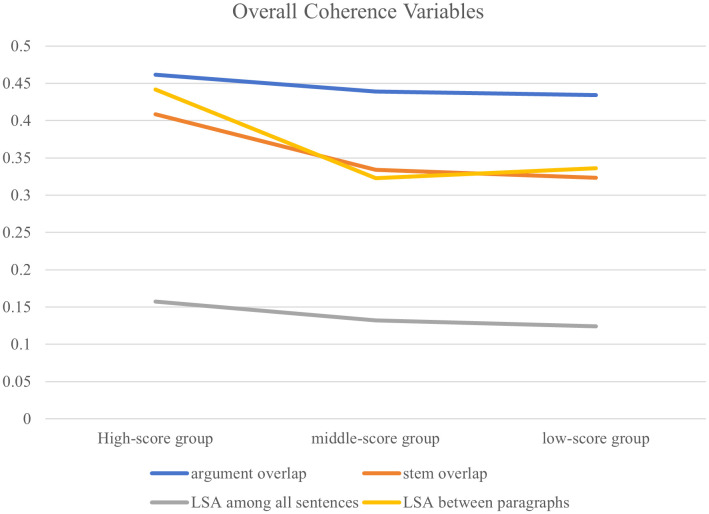
Overall coherence variables.

Regarding overall coherence variables, the high-score group demonstrated the highest values, followed by the middle-score group, with the low-score group exhibiting the lowest values. From the standard deviation of the overall coherence variables within each group, the high-score group presented the minimal standard deviation, the middle-score group presented the moderate standard deviation, and the low-score group presented the maximal standard deviation. The pairwise comparison of overall coherence variable values across three groups indicates that high-score compositions exhibit superior overall coherence with greater internal consistency. The middle-score group ranked second in coherence performance, while low-score compositions presented both the weakest overall coherence and the highest degree of within-group variability. However, the divergence between two adjacent groups is not obvious, which is demonstrated in Fig 2.

#### 4.3.3 Summary.

Comprehensive analysis of local and overall coherence variables reveals an inverse relationship between writing quality and surface cohesion: lower-score texts exhibit elevated values in specific local coherence measures, indicating greater reliance on surface cohesion. Conversely, higher-score writing demonstrates increased overall coherence values, reflecting enhanced semantic depth. These results suggest that students across proficiency levels possess fundamental text awareness and are able to consciously employ text cohesive devices to enhance text consistency. Specifically, high-score writers display superior macro-level composition skills, conceptualizing articles holistically with consistent logical-semantic structuring. In contrast, low-score students think more locally and tend to increase text consistency from a local perspective with more superficial coherence patterns. In this way, students in the high-score group generally have a deeper understanding of text coherence. Students in the low-score group tend to focus on surface text features such as personal pronouns, various conjunctions, and other cohesive devices, resulting in students’ shallow understanding of text coherence. This divergence indicates fundamentally distinct conceptualizations of text coherence: high-proficiency performers prioritize deep semantic integration, whereas low-proficiency performers focus on surface-level connections, and middle-proficiency performers are between these two states.

## 5 Discussion and implications

In order to answer the second research question, this study will discuss students’ linguistic performance in vocabulary, sentences, and text to raise teaching strategies.

### 5.1 Lexis

(1) Lexical dimensions exhibit different sensitivity to writing proficiency.

No significant differences in lexical diversity were observed among the three proficiency groups, which is aligned with Bulté and Housen [79] and Pan and Bao [80]. They have revealed that lexical diversity does not improve significantly as their English proficiency increases. It may be attributed to two reasons: students’ limited vocabulary size and their insufficient understanding of the lexical features of high-quality writing. Meanwhile, the results suggest that lexical diversity alone is not a reliable indicator for distinguishing writing proficiency, as its association with perceived writing quality remains relatively weak. In EFL writing instruction, it is important to raise students’ awareness of the lexical features of proficient writing and to strengthen students’ vocabulary accumulation and application through systematic training.

Lexical sophistication emerged as a crucial indicator of writing quality, demonstrating a relatively strong relationship with perceived writing quality. High-proficiency students employ a greater proportion of low-frequency words in their writings compared to their middle-proficiency counterparts, corroborating studies of Pan and Bao [80] and Wang and Zhou [81]. Conversely, analysis of low-score group compositions revealed frequent reliance on *pinyin* translation, underscoring the need for targeted instruction in the low-frequency vocabulary. Teaching practice should prioritize the English word instruction for low-proficiency students to help them improve their English expressions. Low-frequency word instruction and substitution challenges could be provided to middle-proficiency and high-proficiency students.

Lexical density did not demonstrate a statistically significant association with writing quality, aligning with findings of Engber [4] and Linnarud [82], indicating that the overall information content of a text is not strongly related to its writing quality. As substantiated by Hao et al. [64], higher content-word concentration enhances information quantity conveyed, facilitating more complete meaning transmission. Given that the primary goal of writing is to convey meaning [83], the strategic deployment of lexical density remains relevant for enhancing writing quality. The observed moderate effect size may be attributed to the limited sample size, an issue warranting further investigation with larger samples.

(2) Lexical competence demonstrates non-linear developmental features.

EFL learners at application-oriented universities exhibited a pronounced plateau in the lexical complexity development, which did not develop progressively and linearly. This pattern is in line with the previous studies conducted primarily in research-oriented universities [84,85]. This study suggests the existence of a lexical threshold: learners must attain a certain level of vocabulary knowledge before their writing quality can reach a higher level. The absence of significant differences between the middle-score group and the low-score group across all lexical indicators implies that most learners’ lexical competence in these two groups remains below this threshold. Writing quality can be improved by focusing on the indicator of lexical complexity.

(3) Instructional content should be tailored to learners’ lexical competence.

The findings above address the need to integrate socioculturally relevant vocabulary into curricula to strengthen students’ lexical competence. The limited integration of contemporary sociocultural issues in writing among students is an urgent pedagogical concern. To improve students’ writing and expressive abilities, it is essential to incorporate current affairs lexis systematically. Daily English instructional content should be continuously updated to reflect societal evolution, strategically balancing classical materials with contemporary relevance. Ultimately, cultivating students’ capacity for autonomous language acquisition, particularly through independent input processing and output refinement, should form a core instructional objective.

Higher-rated compositions exhibited greater proportions of low-frequency lexical items, whereas lower-rated texts demonstrated reduced proportions, consistent with the study by Pan and Bao [80]. Meanwhile, it conversely corroborates the findings of Linnarud [82], Vermeer [86], McNamara et al. [71], and Kim [87], with additional evidence from L2 Chinese contexts confirming robust correlations between lexical complexity and writing performance [88,89]. These results imply students’ need to increase their engagement with low-frequency vocabulary through targeted input and production. It is generally believed that high-frequency words are acquired first [90]. Therefore, in the early stages of second language acquisition, there is a quantitative increase in vocabulary size, while in the advanced stages, growth slows down and becomes more qualitative [91]. Consequently, the developmental trajectory of language acquisition necessitates differential lexical distribution in learning materials according to students’ proficiency levels, strategically balancing high-frequency and low-frequency items to optimize acquisition outcomes. Differentiated instructional materials should be provided to students according to their English proficiency levels. For high-proficiency learners, instructional materials may incorporate a higher proportion of low-frequency vocabulary to further enhance lexical sophistication. For middle-proficiency learners, a moderate presence of low-frequency words is recommended, balancing challenge with comprehensibility. For low-proficiency learners, instructional materials should predominantly draw from the most frequent 1,000 and 2,000 word lists, thereby consolidating foundational vocabulary knowledge before progressing to more advanced items.

### 5.2 Syntax

(1) Syntactic competence demonstrates non-linear developmental features.

Syntactic complexity serves as a key dimension distinguishing English writing across proficiency levels and is positively correlated with writing quality. This is manifested in aspects such as the length of linguistic units and syntactic diversity, with various metrics revealing a complex developmental pattern. The study found that students with higher writing proficiency produce longer sentences, T-units, and clauses, and exhibit lower syntactic similarity (i.e., higher syntactic diversity). This aligns with the findings of Ferris [56], whose research indicated that L2 learners with higher proficiency produce significantly more varied sentence structures due to a larger repertoire of syntactic tools at their disposal.

The positive correlation between the length of linguistic units and writing proficiency suggests that more advanced writers are more adept at constructing longer sentences. The differences in sentence length and clause length between the high-score and middle-score groups were statistically significant with a medium-to-large effect size, indicating that these metrics are key syntactic indicators for distinguishing between high-score and middle-score writing. Furthermore, while syntax was more diverse in high-proficiency essays, the middle-score group showed no significant difference from either the high-score or low-score groups. This implies that growth in syntactic diversity is slow with increasing writing proficiency and that this metric alone is insufficient to distinguish writing quality. Regarding clause density, the middle-score group had the highest median value, but the difference from the low-score group was not significant. This suggests that middle-proficiency students tend to use clauses to increase complexity, but their clause application competence deficiency prevents this from effectively enhancing overall writing quality, indicating that merely pursuing a high clause ratio does not necessarily improve writing quality.

Students’ syntactic proficiency appears to develop in a non-linear and cumulative manner. The lack of significant differences between the middle-score and low-score groups across multiple metrics implies that syntactic development from low to intermediate levels is slow and does not yield consistently observable differences. In contrast, the high-score group showed significant differences from both the middle-score and low-score groups in most metrics, indicating a marked leap in syntactic proficiency at the high-proficiency group.

(2) Stratified and progressive syntactic teaching should be implemented.

Regarding pedagogical strategies, teaching objectives should be tiered to strengthen syntactic complexity training. For low-to-intermediate learners, the focus should be on expanding syntactic units and building awareness of syntactic diversity. For intermediate-to-advanced learners, the emphasis should shift towards improving the quality of syntactic production, including training in writing information-intensive clauses, mastering a variety of sentence structures, and enhancing overall sentence quality. In terms of writing assessment and feedback, a dedicated evaluation and feedback system for syntax should be established. This system should prioritize dimensions such as syntactic diversity, the information density of sentences and clauses, and the quality of complex structures. Specific revision suggestions should be provided for issues like repetitive sentence patterns, wordy clauses, and unclear logic found in students’ writing, guiding students to understand the syntactic characteristics of high-quality essays. In teaching practice, curriculum design should incorporate systematic and tiered syntactic training programs. These programs should offer progressive exercises, such as ranging from sentence pattern input and sentence combining to sentence imitation, restructuring, and optimization, to help students at different proficiency levels enhance their syntactic competence. For example, sentence combining exercises could be implemented to gradually increase clausal density for low-proficiency students. Sentence imitation and restructuring tasks could be introduced for middle-proficiency students to focus on clause length and T-unit complexity.

### 5.3 Text

(1) Students’ text coherence awareness shows a consistent tendency with the writing quality.

High-score students demonstrate a stronger ability to conceptualize their writing holistically, showing a more developed sense of text coherence. In contrast, low-score students tend to have more limited thinking, focusing more on local elements to enhance text coherence. A text is formed from a set of sentences guided by the cohesion and coherence among them [92]. Mastery of coherence and cohesion is key to improve the consistency of a text. High-proficiency students can construct text coherence from deeper semantic features, resulting in more logically and semantically consistent texts with stronger overall coherence. These students generally have a deeper understanding of text coherence. However, low-score students often focus on surface text features to achieve coherence, consciously employing cohesive devices such as pronouns and conjunctions. Consequently, the coherence of their texts appears more superficial, and their understanding of text coherence is similarly shallow. These results are related to the EFL writing instruction in China in that teachers tend to equate visible coherence in form with invisible semantic coherence [74]. However, the semantic continuity is the basis of the text coherence, while the cohesive devices in the sentence structure are not enough to construct a cohesive text [32].

(2) Metalinguistic awareness of the text should be addressed to all the students.

In teaching practice, it is necessary to emphasize the significance of holistic conceptualizing in the early stage of writing and to strengthen the practice of overall conceptualizing in the writing. Students with weaker writing performance tend to have a limited focus and lack holistic thinking, necessitating an improvement in their ability to design their compositions as a whole and in their logical planning skills. Therefore, the instruction should initially address all students, with a particular focus on those in the middle-score and low-score groups, to clarify the concept of text and the deep meaning of text coherence. Currently, English instruction often emphasizes the use of surface cohesive devices, such as pronouns and conjunctions, while neglecting the deeper understanding of the text. As a result, students, without a deep understanding of text and coherence, rely excessively on conjunctions and other superficial cohesive devices to create coherence, leading to an overuse of pronouns and conjunctions without significantly improving coherence. Research indicates that there is no obvious relation between the use of cohesive devices and the writing quality, which has a low predictive power for writing performance, and Chinese students tend to overuse these devices [32].

In the future, it is necessary to increase the time and attention devoted to instruction in all these micro dimensions, helping students recognize their significance, and then enhancing students’ autonomous learning in these aspects. It is in line with the research result of Ferris’ study [56]. Ferris [56] held the idea that students need to be encouraged in accurate word selection, diverse sentence usage, and proper use of cohesion and coherence, which may improve students’ using awareness and improve students’ study efficiency and accuracy. Therefore, cultivating students to form a holistic writing cognition is significant to the improvement of their writing performance. For low-proficiency students, instruction should begin with the outlining practice to cultivate holistic text planning and reduce over-reliance on conjunctions and pronouns. Middle-proficiency learners benefit from cohesive device instruction, helping them replace excessive surface markers with logical semantic links. High-proficiency students should engage in the paragraph-level coherence revision, focusing on deep semantic flow rather than superficial cohesion. Across all levels, explicit metalinguistic instruction that distinguishes surface cohesion from deep coherence is essential to break the overuse pattern and foster autonomous writing awareness.

## 6 Conclusion

This study reveals significant variations in linguistic complexity across lexical, syntactic, and textual dimensions among application-oriented university students at high, intermediate, and low proficiency levels. The development of linguistic complexity exhibits non-linear characteristics that align with learners’ writing proficiency, with different linguistic indicators demonstrating differential sensitivity to writing quality.

Pedagogical implications are as follows. First, instructional efforts should prioritize enhancing students’ metalinguistic awareness, fostering their knowledge across all three linguistic dimensions and promoting learner autonomy. Second, writing instruction should be differentiated according to students’ proficiency levels, adopting a focused and progressive approach that addresses their specific developmental needs.

This study advances the existing literature on linguistic complexity in three significant ways. First, this study extends the contextual scope of L2 writing research beyond traditional research-oriented institutions. Second, this study adopts a synchronic stratified design across three proficiency levels and provides a comprehensive understanding of linguistic complexity development. Third, this study employs a comprehensive evaluation framework that integrates lexical, syntactic, and textual levels, thereby offering a more systematic understanding of how different complexity indicators differentially relate to writing proficiency. These contributions collectively inform proficiency-differentiated instructional strategies tailored to the specific needs of application-oriented university EFL learners.

Several limitations of this study should be acknowledged. The primary research focus was on linguistic features; consequently, other potentially influential factors such as cognitive and affective variables were not examined and need future investigation. Additionally, this study takes a single application-oriented university as a case, and the relatively limited sample size in the corpus may pose a limitation for the findings. Future research should expand the sample to incorporate more essays involving students from more application-oriented universities to further explore the developmental patterns of linguistic complexity in English writing in this kind of university.

## Supporting information

S1 File Original data. (XLSX)
